# Does Academic Blogging Enhance Promotion and Tenure? A Survey of US and Canadian Medicine and Pediatric Department Chairs

**DOI:** 10.2196/mededu.4867

**Published:** 2016-06-23

**Authors:** Christian Blake Cameron, Vinay Nair, Manu Varma, Martha Adams, Kenar D Jhaveri, Matthew A Sparks

**Affiliations:** ^1^ Duke University Medical Center Division of Nephrology Durham, NC United States; ^2^ Mount Sinai Medical Center Division of Nephrology New York, NY United States; ^3^ Newark Beth Israel Medical Center Newark, NJ United States; ^4^ Duke University Medical Center Division of General Internal Medicine Durham, NC United States; ^5^ Hofstra Northwell School of Medicine, Northwell Health Division of Kidney Diseases and Hypertension Great Neck, NY United States; ^6^ Duke University and Durham VA Medical Center Division of Nephrology Durham, NC United States

**Keywords:** social media, blogging, promotion, tenure, survey, medicine, pediatrics

## Abstract

**Background:**

Electronic educational (e-learning) technology usage continues to grow. Many medical journals operate companion blogs (an application of e-learning technology) that enable rapid dissemination of scientific knowledge and discourse. Faculty members participating in promotion and tenure academic tracks spend valuable time and effort contributing, editing, and directing these medical journal blogs.

**Objective:**

We sought to understand whether chairs of medicine and pediatric departments acknowledge blog authorship as academic achievement.

**Methods:**

The authors surveyed 267 chairs of US and Canadian medicine and pediatric departments regarding their attitudes toward the role of faculty participation in e-learning and blogging in the promotion and tenure process. The survey completion rate was 22.8% (61/267).

**Results:**

A majority of respondents (87%, 53/61) viewed educational scholarship as either important or very important for promotion. However, only 23% (14/61) perceived importance to faculty effort in producing content for journal-based blogs. If faculty were to participate in blog authorship, 72% (44/61) of surveyed chairs favored involvement in a journal-based versus a society-based or a personal (nonaffiliated) blog. We identified a “favorable group” of chairs (19/59, 32%), who rated leadership roles in e-learning tools as important or very important, and an “unfavorable group” of chairs (40/59, 68%), who rated leadership roles in e-learning tools as somewhat important or not important. The favorable group were more likely to be aware of faculty bloggers within their departments (58%, 11/19 vs 25%, 10/40), viewed serving on editorial boards of e-learning tools more favorably (79%, 15/19 vs 31%, 12/39), and were more likely to value effort spent contributing to journal-based blogs (53%, 10/19 vs 10%, 4/40).

**Conclusions:**

Our findings demonstrate that although the majority of department chairs value educational scholarship, only a minority perceive value in faculty blogging effort.

## Introduction

Widespread adoption of electronic educational (e-learning) technology has fundamentally changed how information is shared and discussed in learning and teaching environments [1]. Examples of e-learning technologies include blogs, social media (Twitter), video and presentation tools (YouTube, Prezi), and online classroom platforms (Sakai, Khan Academy, Coursera). E-learning tools enable rapid, scalable dissemination of information [2]. Blogs in particular have the potential to establish credibility and reach broad audiences in a way that fosters dialog outside of traditional academic “silos” [3]. Scientific usage of blog media and other e-learning technologies may advance innovation and scholarship by shortening the interval between discovery and publication and by soliciting expert, cross-disciplinary feedback throughout the cycle of hypothesis generation, experimental design, execution, data analysis, and interpretation.

Scholarly communication is shifting toward Internet-based media [4]. The field of medicine follows this trend, but lags behind other disciplines [2,5]. Of the medical journals with an impact factor greater than four, approximately 9% have a companion blog [6]. The low level of blogging (blog authorship) in academic medicine may reflect uncertainty among faculty about the role of blogs in relation to traditional forms of scholarship. Prior research has found that faculty work effort and publication patterns are heavily shaped by promotion and tenure requirements, which traditionally emphasize peer-reviewed publications as the benchmark for career advancement [5]. A survey of academic librarians reported that approximately half of respondents believed that a blog post was considered inferior to a traditional peer-reviewed article [7]. However, the posting of scientific information on blogs has the potential to reach a much wider and diverse audience. A recent analysis of page views on a radiology topic (thyroid nodule detection) showed 10-fold greater readership on the blog Radiopaedia.org compared to two traditional journals (*American Journal of Neuroradiology* and the *American Journal of Roentgenology*) [8]. Thus, many academicians have urged promotion and tenure committees to view these activities as bona fide scholarly output when evaluated appropriately [9-11].

We sought to understand whether faculty participation in blogs is acknowledged as academic achievement. Academic blogging can take several forms. In this survey, we focused on participation in medical journal, medical society, and personal (independently authored) blogs. We surveyed all chairs of medicine and pediatric departments in the United States and Canada about their attitudes toward blogging with respect to promotion and tenure. We chose department chairs, as opposed to promotion and tenure committee members, because we believe chairs define the culture for scholarship and promotion within their departments. We hypothesized that chairs who strongly value leadership in e-learning tools would have a favorable attitude toward faculty blogging efforts and recognize these activities as scholarship for the purpose of promotion and tenure.

## Methods

### Recruitment

We invited all chairs of medicine and pediatrics at academic and community-based programs in the United States and Canada to participate in this survey (Multimedia Appendix 1). We obtained email contact information through the Alliance for Academic Medicine and the Association of Medical School Pediatric Department Chairs, with verification and completion of the lists from institutional websites. Institutions were classified as public or private based on the organizational descriptions published on their websites. We received exempted institutional review board approval to conduct the survey from both Duke University Medical Center, Durham, NC (Pro00044769) and Northwell Health, Great Neck, NY (13-107B).

### Survey Administration

A research management team at Duke University reviewed the survey to refine the content. Study data were collected and managed using Research Electronic Data Capture (REDCap) electronic data capture tools hosted at Duke University [12]. REDCap is a secure, Web-based app designed to support data capture for research studies, providing (1) an intuitive interface for validated data entry, (2) audit trails for tracking data manipulation and export procedures, (3) automated export procedures for seamless data downloads to common statistical packages, and (4) procedures for importing data from external sources. Surveys were sent to each of the chairs of medicine and pediatrics via email generated by REDCap in June 2013 (with two additional monthly reminders to nonrespondents). The survey closed in August 2013.

### Evaluation of Outcomes

Respondents that rated the question “How important is a leadership role as a medical director for an e-learning tool, such as editing a journal-based blog” as important or very important were termed the “favorable group” and the respondents that rated the question as somewhat important or not important were termed the “unfavorable group.”

### Statistical Analysis

Analysis was performed to compare the two groups. Items with no response were excluded from analysis. Fisher exact test (two-sided alpha=.05) was used to assess the significance of association between categorical variables.

## Results

### Demographic Data

**Figure 1:**
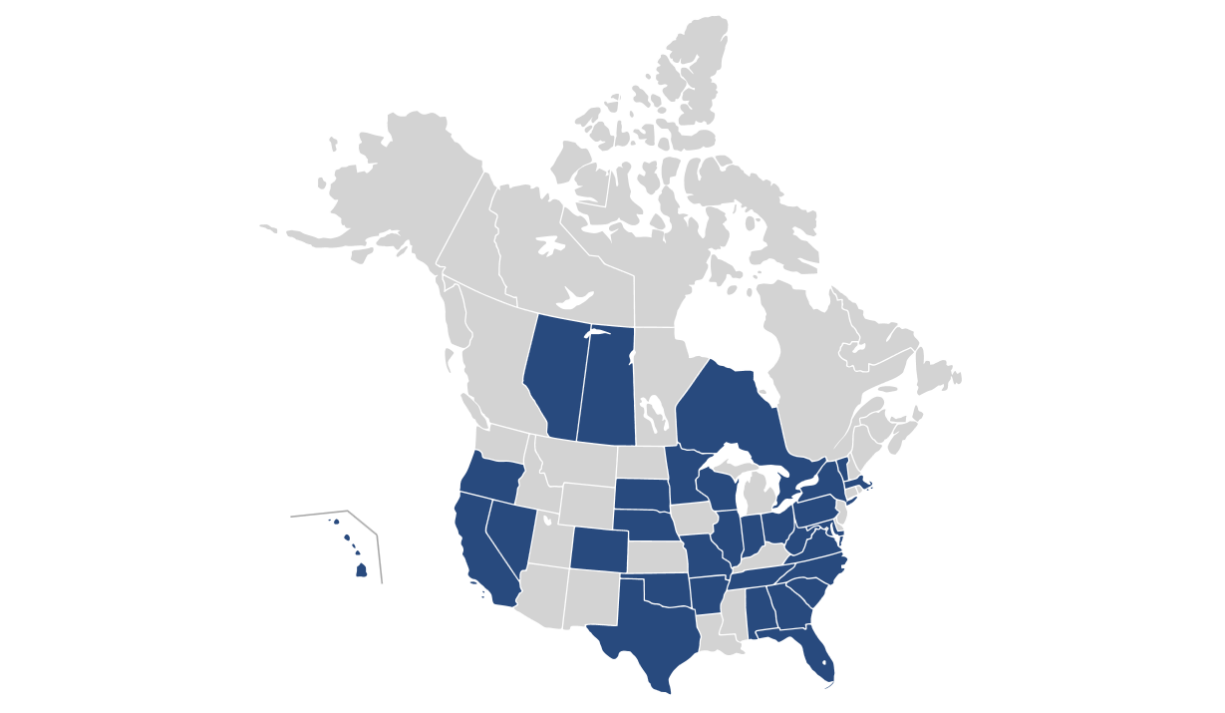
State/province of respondents. State/province (n): Alabama (2), Alberta (1), Arkansas (1), California (1), Colorado (1), Florida (1), Georgia (1), Hawaii (2), Illinois (1), Indiana (2), Maryland (2), Massachusetts (3), Minnesota (1), Missouri (1), Nebraska (2), Nevada (1), New York (4), North Carolina (2), Ohio (6), Oklahoma (1), Ontario (2), Oregon (2), Pennsylvania (1), Saskatchewan (1), South Carolina (2), South Dakota (2), Tennessee (1), Texas (6), Vermont (1), Virginia (4), West Virginia (1), Wisconsin (2), all other states/provinces had zero respondents.

In total, 61 (22.8%) respondents completed the survey from a total of 267 distributed. There was no significant difference in response rate between chairs of public versus private institutions (38/155, 24.5% vs 23/112, 20.5%; *P*=.50). Demographic characteristics of the respondents are shown in Table 1. In all, 62% (38/61) of respondents belonged to public institutions (including one public-private institution) and the remainder were private institutions (Table 1). Of the respondents, 80% (49/61) were male and 55% (33/60) identified their department as pediatrics, with the remainder from medicine. One respondent did not specify a department. Respondents were geographically diverse with representation from 29 states and three Canadian provinces (Figure 1). The majority of respondents (56/61, 92%) reported a clinician-educator track within their departments (Table 1).

**Table 1:** Demographics of respondents (N=61).

Demographic		n (%)
Gender (male)		49 (80)
**Department**		
	Medicine	27/60 (45)
	Pediatrics	33/60 (55)
	No response	1 (2)
**Clinician-educator pathway**		56 (92)
	Medicine	24/56 (43)
	Pediatrics	31/56 (55)
	No response	1/56 (2)
**Institution type**		
	Public	38 (62)
	Private	23 (38)

### Attitudes and Opinions

**Figure 2:**
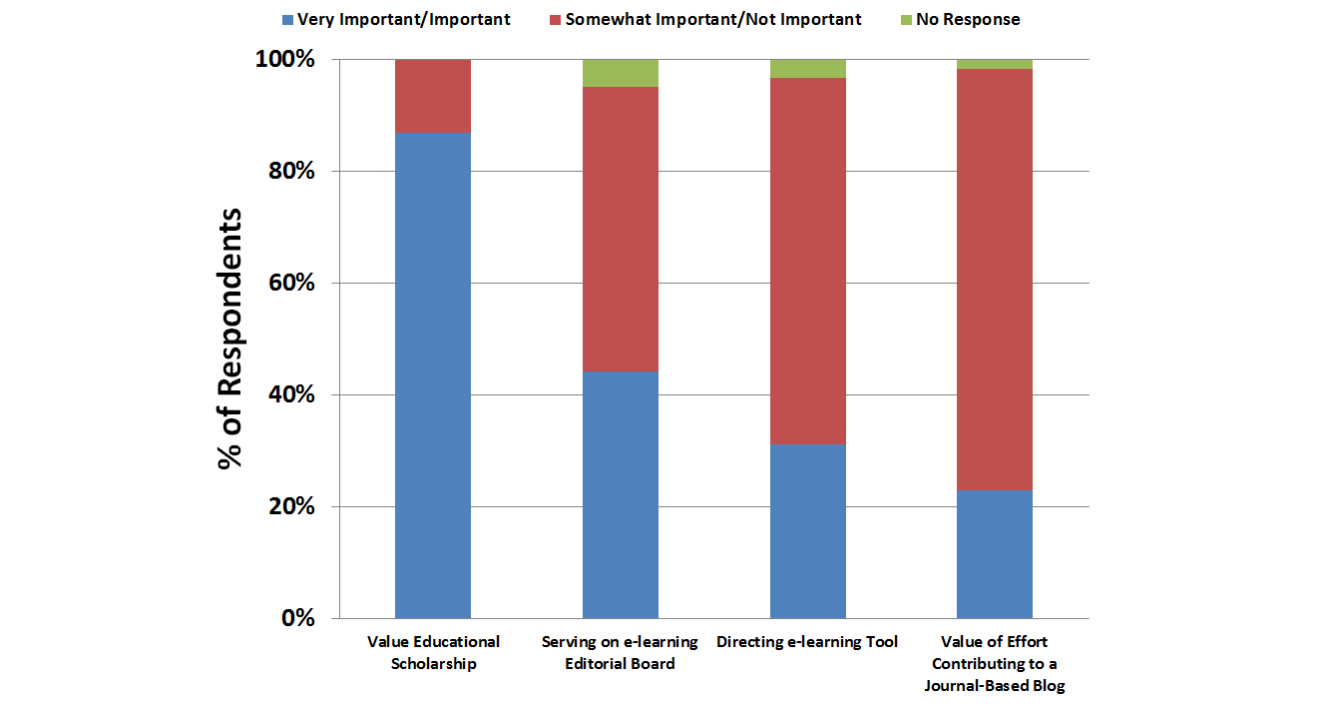
Questionnaire items addressing importance of educational scholarship, leadership in e-learning tools, and valuation of blogging effort.

**Figure 3:**
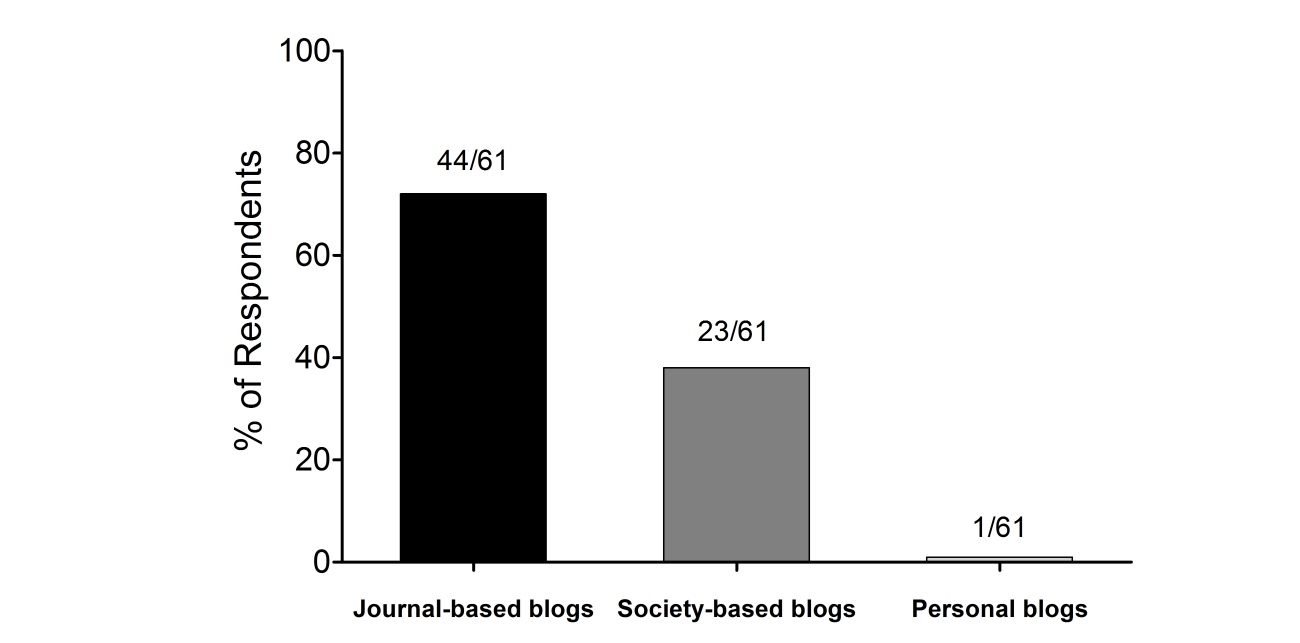
If your faculty member is involved in contributing to an academic blog, which would you value most?

A total of 87% (53/61) of respondents viewed educational scholarship as either important or very important for promotion (Figure 2). However, only 31% (19/61) felt that faculty leadership of an e-learning tool such as a journal-based blog was important or very important for consideration of promotion and tenure. In all, 44% (27/61) felt that serving on an e-learning editorial board was important or very important for promotion and tenure; 23% (14/61) perceived value to faculty effort in producing content for a journal-based blog as important or very important (Figure 2). The vast majority of respondents indicated that they would prefer a faculty member’s contribution to a journal-based blog (44/61, 72%) as compared with professional society-based (23/61, 38%) or unaffiliated personal (1/61, 2%) blogs (Figure 3).

In all, 34% (21/61) of respondents were aware of faculty within their departments that were involved in contributing to a journal-based blog and 43% (26/60) designated a specific area in the promotion and tenure application for reporting participation in journal-based blogs or e-learning tools. Although the majority (49/58, 84%) felt that journal-based blogs disseminate medical knowledge, only 25% (14/56) believed that most journal-based blogs were peer reviewed by editors.

Among respondents who rated leadership roles in e-learning tools (eg, directorship of a journal-based blog) as important or very important (the favorable group; n=19/59, 32%), as compared to somewhat important or not important (the unfavorable group; n=40/59, 68%), there was no significant difference in gender or the presence of a clinician-educator track. There was a trend toward a more favorable attitude by medicine chairs versus pediatric chairs (12/19, 63% vs 14/39, 36%; *P*=.09). However, the chairs in the favorable group were more likely to be aware of faculty bloggers within their departments (11/19, 58% vs 10/40, 25%; *P*=.02; Table 2). These respondents viewed serving on editorial boards of e-learning tools more favorably (15/19, 79% vs 12/39, 31%; *P*<.001; Table 2) and were more likely to value effort spent contributing to journal-based blogs (10/19, 53% vs 4/40, 10%; *P*<.001; Table 2). There was no difference in recognition of blogging or e-learning on promotion and tenure applications, belief in peer review of journal-based blogs, belief that journal-based blogs disseminate medical knowledge, or preference for contributions to any particular type of blog.

**Table 2:** Comparison of attitudes categorized by favorable versus unfavorable chairs.

Characteristic	Overall response, n (%) (n=59)^a^	Favorable group,^b^ n (%) (n=19)	Unfavorable group,^c^ n (%) (n=40)	*P*
Male	47 (80)	14 (74)	33 (83)	.50
Medicine department	26/58 (45)	12 (63)	14/39 (36)	.09
Have a clinician-educator pathway	54 (92)	19 (100)	35 (88)	.17
Educational scholarship important or very important for promotion	52 (88)	18 (95)	34 (85)	.41
Serving on editorial boards of e-learning tools important or very important	27/58 (47)	15 (79)	12/39 (31)	<.001
Aware of faculty bloggers in department	21 (36)	11 (58)	10 (25)	.02
Promotion and tenure form has field for blogging and e-learning	26/58 (45)	9 (47)	17/39 (44)	>.99
Effort contributing to a journal-based blog important or very important	14 (24)	10 (53)	4 (10)	<.001
Believe most journal-based blogs are peer reviewed	13/55 (24)	6/18 (33)	7/37 (19)	.31
Believe journal-based blogs disseminate knowledge	48/57 (84)	17 (89)	31/38 (82)	.70
Prefer contributions to journal blogs	43 (73)	13 (68)	30 (75)	.76
Prefer contribution to society blogs	22 (37)	10 (53)	12 (30)	.15
Prefer contribution to personal blogs	1 (2)	0 (0)	1 (3)	>.99

^a^ Respondents who failed to answer e-learning leadership question were removed from analysis. Likewise, respondents who answered leadership but did not answer question as outlined on the rows were not included in analysis.

^b^ E-learning leadership considered very important/important.

^c^ E-learning leadership considered somewhat important/not important.

## Discussion

### Principal Results

In our survey of chairs of medicine and pediatric departments in the United States and Canada, we found that the vast majority valued participation in educational scholarship for promotion in tenure. However, it appears that participation in e-learning activities, as director, board member, or contributor of a blog, was generally viewed less favorably than other traditional publication activities. If faculty chose to participate in blogging, we found that a majority of chairs of medicine and pediatric departments preferred that they do so with journal-based blogs as compared to society and independent personal blogs.

This survey showed that chairs who thought e-learning leadership was very important or important to promotion and tenure (the favorable group) were more likely to be aware of a faculty member engaged in medical blogging, as compared to chairs who indicated that e-learning leadership role was somewhat important or not important (unfavorable group). The favorable group was also more likely to value e-learning for promotion and tenure, faculty service to an editorial board of an e-learning app, and contributions to journal-based blog. The attitudes of the favorable group are similar to a survey of US medical school promotion and tenure committee chairs on attitudes toward e-learning as scholarly activity. A vast majority (76%) recognized e-learning as a meaningful contribution to scholarship and valued output that changed learner outcome [13].

### Comparison With Prior Work

Blogging has emerged as a new medium to disseminate scientific findings, spark academic dialog, and complement existing postpublication peer-review mechanisms (eg, journal editorials or letters to the editor). Online resources geared toward critical appraisal of scientific and medical literature have proliferated in the last 5 to 10 years [14], garnering unprecedented readership and complementing traditional scientific communication [15]. However, it is yet to be determined whether the time and effort that faculty spend on these communication activities contributes to promotion and tenure [16]. Our aim was to understand the perceptions and attitudes of medicine and pediatric department chairs toward faculty participation in medical blogs and its influence on academic advancement. We chose to survey department chairs, instead of promotion and tenure committee chairs, because we believe that these individuals (1) possess knowledge of faculty activities and institutional practices, and (2) shape the culture, guidelines, and processes for promotion within their departments. Furthermore, a survey of promotion and tenure committee chairs has already been performed [13]. Physicians and academicians are increasingly utilizing blogs and other e-learning technologies for scientific communication, and we believe it is necessary to recognize these efforts.

### Limitations

This survey has several limitations. The relatively low response rate (22.8%, 61/267) of surveyed chairs may not be representative of the entire group’s attitudes toward e-learning. It is possible that polarization of attitudes among respondents led to sampling bias. We did not ask chairs to evaluate e-learning and blogging participation against specific comparators, such as research grants or publication in peer-reviewed journals. In addition, respondents’ understanding of personal (unaffiliated) medical blogs may have been conflated with nonmedical “hobby” blogs. It is also possible that opinions of department chairs, as opposed to promotion and committee member chairs, are not representative of what is ultimately deemed acceptable scholarly output. However, as previously stated, we intentionally targeted department chairs because we believe they are aware of the scholarly activities their faculty members engage in and help determine the relative importance of such activities. Additionally, we chose to dichotomize the survey respondents as favorable and unfavorable based on their answer to one question (“How important is a leadership role as a medical director for an e-learning tool, such as editing a journal-based blog?”). Conceptual similarities between this question and the other questions could have led to results that are endogenous to our classification.

### Conclusions

The findings from our survey indicate that, although academic chairs hold positive views toward educational scholarship, participation by faculty in academic blogs is regarded lukewarmly. Encouragingly for faculty bloggers, chairs that did favorably view e-learning educational output by faculty were indeed more aware of faculty members’ blog involvement. Therefore, it is possible that raising awareness about the rigor and reach of e-learning, particularly in regards to journal-based blogs, could lead to greater recognition of e-learning participation in the promotion and tenure process. As more medical societies and journals develop e-learning programs that shift discourse to the digital space, academic institutions, department chairs, and promotion and tenure committees should recognize faculty that contribute significant scholarship through these media.

## Appendix

Multimedia Appendix 1 Survey participation letter.
