# Supplementary material for: Does Academic Blogging Enhance Promotion and Tenure? A Survey of US and Canadian Medicine and Pediatric Department Chairs
Source: JMIR Med Educ. 2016 Jun 23;2(1):e10. doi: 10.2196/mededu.4867 (PMC5041355; doi:10.2196/mededu.4867)
Supplement: Multimedia Appendix 1 [file mededu_v2i1e10_app1.pdf]

Perspectives on academic promotion for e-learning tools and social media work

Dear Academic Chair:

We are writing this letter to request that you take part in this quick 5 minute survey to better understand how contribution to medical journal-based blogs affects promotion and tenure. An increasing number of medical journals are starting blogs (web journals) to both promote and enhance journal content. Popular examples include the NOW@NEJM (http://blogs.nejm.org/now/) and the news@JAMA (http://newsatjama.jama.com/). As such, the number of physicians and scientists authoring and editing the journal-based blogs are increasing.

We are interested in understanding how a faculty member's effort in creating content for journal-based blogs affects their promotion at your institution. The results of this survey can assist in the development of future guidelines to promotion and tenure committees that can better inform them with objective criteria to make more informed decisions.

Participation is voluntary. If you would prefer not to participate, simply do not complete the attached survey. If you agree to participate, please complete the attached survey. Your responses are anonymous; do not put your name or other identifying information on this survey. We ask that you try to answer all questions. However, if there are any questions that you would prefer to skip, simply leave the answer blank.

This research has been reviewed by the Institutional Review Board (IRB) at the North Shore LIJ Health System and Duke University Medical Center (Pro00044769) and is deemed exempt (45CFT46.101(b)). If you have any questions about your rights as a participant, or if you feel that your rights have been violated, please contact the North Shore LIJ Health System IRB at 516-562-3101.

Please contact Dr. Kenar Jhaveri for any questions regarding the survey at kjhaveri@nshs.edu

Sincerely,

Kenar D. Jhaveri, MD
Hofstra NSLIJ School of Medicine

Vinay Nair, DO
Icahn School of Medicine at Mount Sinai

Manu Varma
Lake Erie College of Osteopathic Medicine

Martha Adams, MD
Duke University Medical Center

Matthew A. Sparks, MD
Duke University Medical Center

1) We are interested to know the region of the country where you are the Chair. Please indicate your state.

- ☐ Alabama
- ☐ Alaska
- ☐ Arizona
- ☐ Arkansas
- ☐ California
- ☐ Colorado
- ☐ Connecticut
- ☐ Delaware
- ☐ District of Columbia
- ☐ Florida
- ☐ Georgia
- ☐ Guam
- ☐ Hawaii
- ☐ Idaho
- ☐ Illinois
- ☐ Indiana
- ☐ Iowa
- ☐ Kansas
- ☐ Kentucky
- ☐ Louisiana
- ☐ Maine
- ☐ Maryland
- ☐ Massachusetts
- ☐ Michigan
- ☐ Minnesota
- ☐ Mississippi
- ☐ Missouri
- ☐ Montana
- ☐ Nebraska
- ☐ Nevada
- ☐ New Hampshire
- ☐ New Jersey
- ☐ New Mexico
- ☐ New York
- ☐ North Carolina
- ☐ North Dakota
- ☐ Ohio
- ☐ Oklahoma
- ☐ Oregon
- ☐ Pennsylvania
- ☐ Rhode Island
- ☐ South Carolina
- ☐ South Dakota
- ☐ Tennessee
- ☐ Texas
- ☐ Utah
- ☐ Vermont
- ☐ Virginia
- ☐ Washington
- ☐ West Virginia
- ☐ Wisconsin
- ☐ Wyoming

(Pick Your State)

2) What is your gender?

- ☐ Male
- ☐ Female

3) Please indicate your department.

- ☐ Medicine
- ☐ Pediatrics

4) Do you have a "clinician-educator" track in your program?

- ☐ Yes
- ☐ No

5) How important is educational scholarship to a candidate's opportunity for promotion, e.g., publishing, creating enduring materials, and performing other scholarly work in education?

- ☐ Very important
- ☐ Important
- ☐ Somewhat important
- ☐ Not important

6) How important is serving on editorial boards of medical e-learning groups and websites?

☐ Very important
☐ Important
☐ Somewhat important
☐ Not important

7) How important is a leadership role as a medical director for an e-learning tool, such as editing a journal-based blog?

☐ Very important
☐ Important
☐ Somewhat important
☐ Not important

8) Are you aware of any faculty members in your department who are involved in journal-based blogs?

☐ Yes
☐ No

9) At your institution is there a specific location in the application for promotion and tenure where a physician can indicate involvement in journal-based blogs or e-learning tools?

☐ Yes
☐ No

10) Please rate how you perceive the value of the effort a faculty member contributes to writing a journal-based blog.

☐ Very Important
☐ Important
☐ Somewhat important
☐ Not important

11) True or False: "The majority of journal-based blogs are edited and peer reviewed with the journal editors."

☐ True
☐ False

12) True of False: "Journal-based blog content helps in the dissemination of medical knowledge."

☐ True
☐ False

13) If your faculty member is involved in contributing to an academic blog, which would you value most? (Check all that apply).

☐ Journal-based blogs
☐ Society-based blogs
☐ Personal blogs

14) Any other comments?

____
